# Probiotic *Lactobacillus rhamnosus* GR-1 supplementation attenuates Pb-induced learning and memory deficits by reshaping the gut microbiota

**DOI:** 10.3389/fnut.2022.934118

**Published:** 2022-07-19

**Authors:** Xiaozhen Gu, Nanxi Bi, Tian Wang, Chengqing Huang, Rongrong Wang, Yi Xu, Hui-Li Wang

**Affiliations:** ^1^Engineering Research Center of Bio-process, Ministry of Education, Hefei University of Technology, Hefei, China; ^2^School of Food and Biological Engineering, Hefei University of Technology, Hefei, China

**Keywords:** Pb exposure, learning and memory deficits, *Lactobacillus rhamnosus*, G-CSF, gut microbiota

## Abstract

Lead (Pb) exposure during early life has been associated with an increased risk of neurodevelopmental disorders, including learning and memory deficits. The intestinal flora, *via* the microbiome–gut–brain axis, could play a significant role in the nervous system. However, the effects of probiotics on ameliorating Pb-induced learning and memory deficits are still unclear. In this study, we showed that adolescent Pb exposure (150 ppm) for 2 months impaired spatial learning and memory ability, accompanied by the decreasing diversity of gut microbiota, and the decreasing abundance of *Lactobacillu*s at the genus level. Surprisingly, administration of the *Lactobacillus rhamnosus* GR-1 (10^10^ organisms/rat/day), not *L. rhamnosus* LGG or *Lactobacillus reuteri* RC-14, reversed learning and memory deficits induced by Pb exposure. Meanwhile, administration of the *L. rhamnosus* GR-1 increased the diversity of the gut microbiota composition and partially normalized the genus level of *Lactobacillus*, *Parabacteroides*, *Enterococcus*, and *Akkermansia* in Pb-exposed rats. Notably, supplementation of *L. rhamnosus* GR-1 decreased the gut permeability of Pb-exposed rats, reduced proinflammatory cytokines [interleukin-1β (IL-1β) and IL-6] expression, and promoted anti-inflammatory cytokines [granulocyte colony-stimulating factor (G-CSF)] expression. Interestingly, neural cell treatment with G-CSF rescued Pb-induced neurotoxicity. In general, *L. rhamnosus* GR-1 supplementation recovered the Pb-induced loss of intestinal bacteria (*Lactobacillus*), which may have reversed the damage to learning and memory ability. Collectively, our findings demonstrate an unexpectedly pivotal role of *L. rhamnosus* GR-1 in Pb-induced cognitive deficits and identify a potential probiotic therapy for cognitive dysfunction during early life.

## Highlights

- *Lactobacillus rhamnosus* GR-1 alleviates Pb-induced learning and memory deficits by remodeling the gut microbiota.
- *Lactobacillus rhamnosus* GR-1 repairs intestinal damage and reduces hippocampal microgliosis and proinflammatory cytokines of Pb-exposed rats.
- *Lactobacillus rhamnosus* GR-1 rescues the Pb-induced neurotoxicology by promoting anti-inflammatory cytokines G-CSF expression.

## Introduction

Childhood lead (Pb) exposure is considered a tremendous public health challenge in the world. Compelling studies suggested an association between Pb exposure and cognitive deficits. Such detrimental effects have been found in people across their lifespan, meaning that Pb exposure earlier in life may increase the risk for later dementia (1, 2).

In recent decades, microbiology and neuroscience have become ever more entwined (3). The concept of the microbiota–gut–brain axis shows that the resident microbiota can exert considerable influence over host behavior (4). Previous studies have shown that Pb exposure reduced the expression of intestinal tight junction protein and disrupted gut barrier integrity (5). Recent evidence has linked probiotics with Pb-induced cognitive impairment by remodeling gut microbiota of Pb-exposed rats (6). However, we still lack a complete understanding of how specific strains may influence the gut–brain axis and repair Pb-induced cognitive impairment.

Supplementation of probiotics is essential for maintaining human intestinal health. Recent studies have found that probiotics can treat enteritis, diabetes, hypertension, and hyperlipidemia (7). *Lactobacillus rhamnosus*, a Gram-positive bacterium belonging to the *Lactobacillus* genus, has a high intestinal adhesion rate and strong colonization ability (8, 9). It has the biological characteristics of acid resistance, bile salt resistance, and inhibition of the proliferation of harmful bacteria. After colonization in the intestine, *L. rhamnosus* can attach to intestinal epithelial cells and act as a biological barrier. It can also regulate the balance of host intestinal flora, prevent the occurrence of diarrhea and dental caries, and improve body immunity (10). Studies on whether *L. rhamnosus* can repair Pb-induced memory impairment are lacking. Therefore, the effects and mechanisms of *L. rhamnosus* on cognitive deficits are yet to be studied.

In this study, we sought to identify the mechanism by which a given bacterial strain could reverse Pb-induced cognitive deficits. Genetics, metagenomics, immunohistochemistry, electrophysiology, and Morris water maze (MWM) assays were used to dissect how *L. rhamnosus* GR-1 impacts cognitive function. Notably, oral treatment with *L. rhamnosus* GR-1 attenuated Pb-induced learning and memory deficits by restoring intestinal permeability of the colon and microgliosis in the hippocampus. Our findings suggest that targeting the microbiome may represent an approach for targeting the individual with Pb-induced cognitive deficits, realizing the potential for developing microbiota-based therapeutic strategies for brain disorders.

## Materials and methods

### Animals and study design

All the animals [Sprague–Dawley (SD) rats] needed for the experiment were purchased from the Laboratory Animal Center of Anhui Medical University. All rats had free accessibility for water and food and were housed under specific pathogen-free conditions (light/dark alternate for 12 h, 20–25°C, 50–60% air humidity). All experimental procedures were approved by the Institutional Animal Care and Use Committee of Hefei University of Technology, China. The female pups postnatal day 21 (PND 21) were randomly selected after weaning in each group and subjected to subsequent trials. Pb acetate (150 ppm) was administered *ad libitum* in drinking water directly after weaning (PND 21) till sacrifice (PND 60). The Pb-exposed model was referenced in the previous studies (11).

The *L. rhamnosus* GR-1 we used in this study were separated from UMETA probiotics produced by BGI Company, China. For probiotic treatment, *L. rhamnosus* GR-1 were supplied at a dosage of 10^10^ organisms/rat/day for 0.2 ml in sterilized water through gavage from PND 21 till sacrifice (the same period with Pb exposure) (6).

### Antibodies and reagents

Anti-IBA1, anti-TJP1, and anti-OCLN were purchased from ProteinTech, Wuhan, China. Anti-P-synapsin-1, VGlut1, NMDAR1, and NMDAR2B were purchased from Abcam, Shanghai, China. 4,6-Diamidino-2-phenylindole (DAPI; #C0060) was purchased from Solarbio, Beijing, China.

### Behavioral assessment

Morris water maze test was conducted according to our previous study (12). The moving tracks were video-recorded and automatically scored using the Smart tracking software (ANY-maze, Stoelting, Shanghai, China). The speed, platform-crossing times, time spent on the target quadrant, and total moving distances were recorded and analyzed.

### Golgi–Cox staining

Golgi–Cox staining was conducted according to our previous study (13). At PND 70, the hippocampus was rapidly collected and Golgi–Cox staining was performed in order to analyze spinal density in the CA1 and dentate gyrus (DG) regions of the hippocampus, as previously described (13). Specifically, the dendritic sections were imaged with a Nikon microscope (Eclipse 80i, Nikon, Tokyo, Japan).

The double-blind method was used for selection and statistics. The spines in the secondary and tertiary branches were counted. The density of dendritic spines per 10 μm was calculated using MATLAB. A total of 50 granular or pyramidal cells were randomly selected from each group.

### Microbiome analysis

The fresh feces from adult female rats (PND 60) were collected, homogenized, and subjected to DNA extraction using the HiPure Stool DNA Kit B (Magen, Guangzhou, China) according to the instructions of the manufacturer. Subsequently, 1 ng/μl of DNA was subjected to 16SV4 rRNA amplification. The primer pair used in V4 amplification is 515F (5′-GTGYCAGCMGCCGCGGTAA-3′) and 806R (5′-GGACTACNVGGGTWTCTAA-3′). The PCR products were extracted and purified using the GeneJET Gel Extraction Kit (Thermo Fisher Scientific, Beijing, China) according to the instructions of the manufacturer.

The library was constructed using the Ion Plus Fragment Library Kit (Thermo Fisher Scientific, Beijing, China). Following the Qubit quantification, the pooled amplicons were subjected to IonS5TMXL for sequencing. Data analysis was conducted according to our previous study (6).

### Western blot

The protein levels of P-synapsin-1, VGlut1, NMDAR1, and NMDAR2B were detected, respectively. Densitometry was assessed with the ImageJ software, with protein quantity normalized to GAPDH. The Western blot assay was conducted according to our previous study (1).

### Immunohistochemistry

After > 24 h of fixation in 4% polyformaldehyde, the brain tissues of all groups were dehydrated with a sucrose solution. The brain was wrapped in the Tissue-Tek O.C.T. Compound (Radnor, PA, United States) and immediately frozen to −20°C. The fixed tissue was sectioned into coronal sections with a width of 30 μm using a slicer cryostat. A 0.2% Triton X-100 solution was added for 4 h to make the tissue sections permeable. After blocking with 10% goat serum for 4 h, the brain slices were incubated with rabbit anti-IBA1 antibody overnight. Goat anti-rabbit IgG (H + L) secondary antibody (Boster, Wuhan, China; 1/50) was used to label the IBAI antibody at 4°C for 1 h. Fluorescent imaging was performed using the confocal laser scanning microscope.

### H&E staining

After the behavior test, the hematoxylin and eosin (H&E) staining was performed as described in detail previously (14). After the rats were anesthetized, the colon tissue was soaked in fixed with 4% paraformaldehyde solution, then rinsed with phosphate-buffered saline (PBS), gradient dehydration with 75, 80, 95, and 100% ethanol. After removing all ethanol, the tissue was paraffin-embedded. The tissue sections were stained with H&E and observed with a fluorescence light microscope.

### Fecal microbiota transplantation

Specifically, fresh fecal samples were collected from Pb and Pb + GR-1, respectively. The collected feces were mixed in a 1:4 ratio in a sterile solution (1 × PBS: 80% glycerol, ratio 1:1), centrifuged at 1,000 × *g* for 3 min at 4°C, and the supernatant was collected. Fecal transplantation (200 μl supernatant/rat/day) was performed at PND 21 of Pb-exposed rats by oral gavage for 4 weeks (15).

### Enzyme-linked immunosorbent assay

Levels of cytokines [interleukin (IL)-1β, IL-6, IL-10, tumor necrosis factor-α (TNF-α), and granulocyte colony-stimulating factor (G-CSF)] were all measured using ELISA kits (mlbio, Wuhan, China), according to the instructions of the manufacturer.

### Quantitative RT-PCR analysis

The total RNA extraction and reverse transcription assay were performed according to our previous study (6). The primers used in this study are listed in Supplementary Table 1. The β-actin was used as an internal control.

### Quantification of neurite outgrowth in PC12 cells

Neurite outgrowth is a key process during neuronal migration and differentiation. When fully differentiated through axon and dendrite elongation, this unique morphology allows neurons to achieve precise connectivity between appropriate sets of neurons, which is crucial for the proper functioning of the nervous system (16). Primary and secondary neurite quantifications were counted as previously described (17).

### Primary neuronal culture electrophysiological recordings

Whole-cell patch-clamp recordings were performed according to the previous study (18, 19). The cultured primary hippocampus neurons were treated with Pb acetate (5 μm) and G-CSF (50 ng/ml) for 48 h. Then, cultured primary hippocampus neurons at days in vitro (DIV) 14 were performed using whole-cell patch-clamp recordings.

### Statistical analysis

Graph data are presented as mean ± SEM. Statistical analysis was performed using the SPSS software. When the experimental setting contained four groups (Ctrl, Pb, Pb + GR-1, GR-1), two-way ANOVA was used to perform a single endpoint analysis. When the experiment was designed in two groups, *t*-test was used to perform a single endpoint analysis. The data meet the assumptions for the specific statistical test we chose, and the tests were automatically adjusted when variances among the groups differed. *p* ≤ 0.05 was regarded as statistically significant.

## Results

### Species-specific role for the administration of *Lactobacillus rhamnosus* GR-1 alleviated Pb-induced learning and memory deficits

**FIGURE 1:**
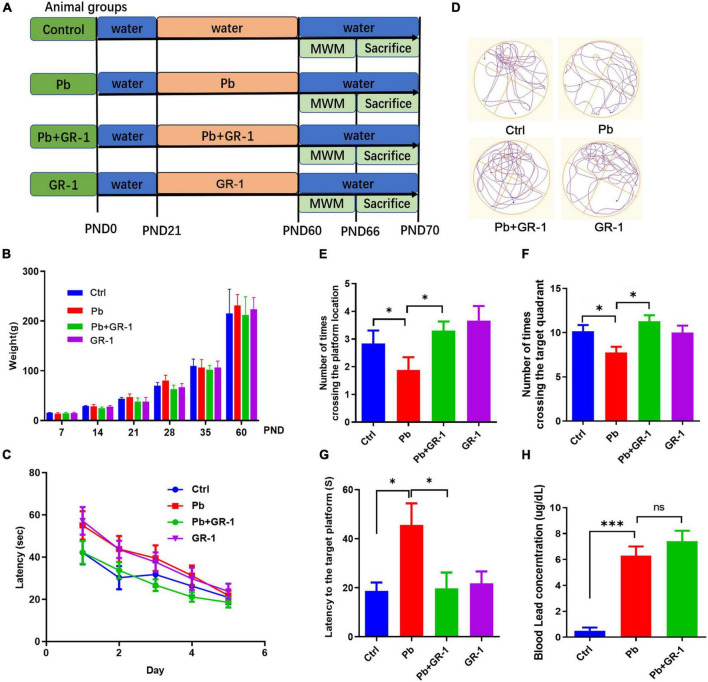
Administration of *Lactobacillus rhamnosus* GR-1 alleviated Pb-induced learning and memory deficits. **(A)** Scheme of treatment and behavioral tests in 8-month-old SD rats receiving sham or *Lactobacillus rhamnosus* GR-1 by gavage daily from postnatal day 21 (PND 21) to PND 60. **(B)** The weight of the rats was recorded throughout the trial period. **(C)** The escape latencies of the four groups over five consecutive training days. **(D)** Representative swimming paths of Ctrl (*n* = 9), Pb (*n* = 9) or Pb + GR-1 (*n* = 9), and GR-1 (*n* = 9) on the test day. **(E–G)** Average crossing number over the platform-site **(E)**, number of times crossing the target quadrant **(F)**, and latency of the first target-site crossover during the test day **(G)**. **(H)** The blood Pb concentration of the indicated groups. Data are shown as mean ± SEM. *p < 0.05, ^***^p < 0.001.

Recent evidence suggests that a complex probiotic containing *Bifidobacterium longum*, *L. rhamnosus*, and *Streptococcus thermophilus* alleviated Pb-induced cognitive impairment by remodeling gut microbiota of Pb-exposed rats (6). Next, we wondered that only *Lactobacillus* treatment can restore cognitive function in Pb-exposed rats. To test this hypothesis, we introduced *L. rhamnosus* GR-1 through intragastric gavage for 4 weeks of Pb-exposed rats after weaning, after which MWM behavior was tested (Figure 1A). There was no significant difference in body weight (BW) between the indicated groups (Figure 1B). To assess spatial learning and memory ability, we performed the MWM test by recording the number of times crossing the target platform, the distance moved, and velocity during the time spent in the MWM water tank. The results showed that *L. rhamnosus* GR-1 reduced the probing time to the target platform and increased the number of times crossing the target platform and quadrant of Pb-exposed rats (Figures 1C–G), indicating that the administration of *L. rhamnosus* GR-1 restored the Pb-induced learning and memory deficits. Importantly, similar treatment with other *Lactobacillus* species, such as *L. rhamnosus* LGG, *Lactobacillus reuteri* RC-14, failed to rescue cognitive behaviors in Pb-exposed rats (Supplementary Figures 1, 2). It indicated that ameliorating the deficient cognitive behavior is a strain-specific role in administering *L. rhamnosus* GR-1. Besides, the *L. rhamnosus* GR-1 used in this study failed to reduce the concentration of Pb in the blood, indicating that *L. rhamnosus* GR-1 did not exert its probiotic function on cognitive by reducing the blood Pb concentration (Figure 1H).

To further investigate the critical components of *L. rhamnosus* GR-1 that restored Pb-induced learning and memory deficits, Pb-exposed rats were treated with either culture medium supernatant of *L. rhamnosus* GR-1 or heat-killed *L. rhamnosus* GR-1 (80°C for 20 min) by oral gavage. The MWM test found that neither culture medium supernatant of *L. rhamnosus* GR-1 nor heat-killed *L. rhamnosus* GR-1 restored the cognitive behavior by MWM test (Supplementary Figure 3). The data indicated that *L. rhamnosus* GR-1 specifically rescued Pb-induced learning and memory deficits depending on the activity of *L. rhamnosus* GR-1.

### *Lactobacillus rhamnosus* GR-1 supplementation rescued the morphological abnormalities of dendritic spines and the related protein expressions in synaptic transmission in the hippocampus after Pb exposure

**FIGURE 2:**
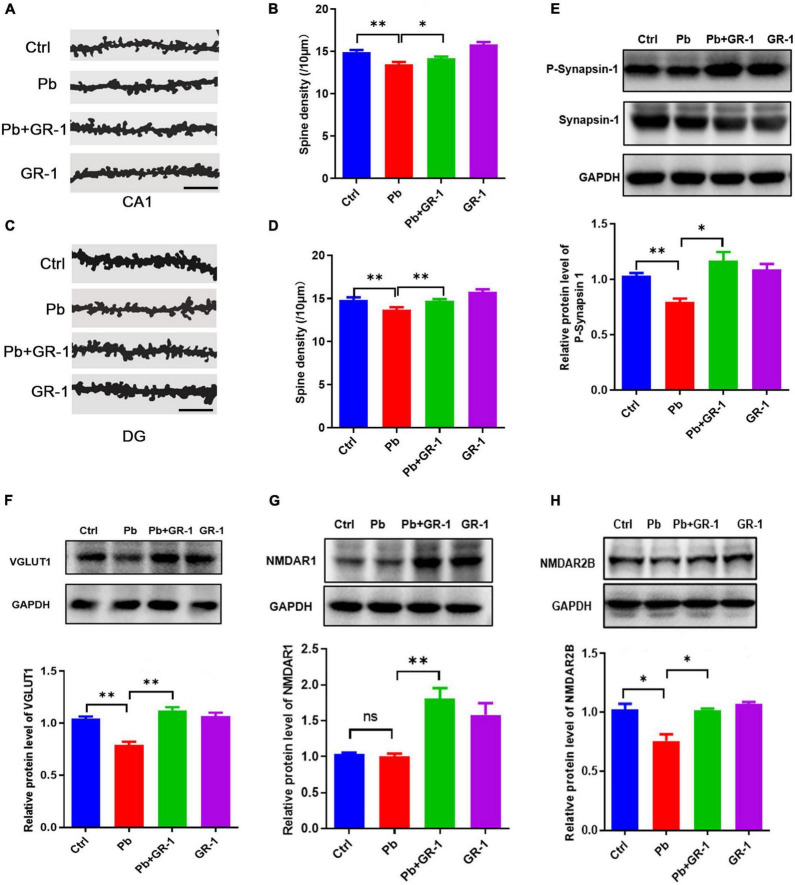
*Lactobacillus rhamnosus* GR-1 supplementation rescued the morphological abnormalities of dendritic spines in the hippocampus. **(A,B)** Representative images **(A)** and quantification of spine densities are shown in the region of CA1 **(B)**. Scale bar = 10 μm, *n* = 50. **(C,D)** Representative images **(C)** and quantification of spine densities are shown in the region of DG **(D)**. Scale bar = 10 μm, *n* = 50. **(E–H)** Immunoblots and quantification of protein levels of P-synapsin-1, VGlut1, NMDAR1, and NMDAR2B upon the administration of *Lactobacillus rhamnosus* GR-1 of Pb-exposed rats. *n* = 3. Data are shown as mean ± SEM. *p < 0.05, ^**^p < 0.01.

The morphology and number of dendritic spines in the hippocampus are closely related to the formation of learning and memory (20). We next wondered whether *L. rhamnosus* GR-1 treatment, which restores learning and memory deficits in Pb-exposed rats, would also rescue hippocampus neuron spine structure. The Golgi–Cox staining revealed that *L. rhamnosus* GR-1 treatment rescued the spine density of hippocampal neurons in Pb-exposed rats (Figures 2A–D). The related protein expressions in synaptic transmission were detected, including P-synapsin-1, VGlut1, NMDAR1, and NMDAR2B. Indeed, we found that *L. rhamnosus* GR-1 treatment reversed the P-synapsin-1, VGlut1, and NMDAR2B expressions of Pb-exposed rats (Figures 2E–H). Besides, the administration of *L. rhamnosus* GR-1 significantly upregulated NMDAR1 expression compared to the control group. Taken together, our results indicated that *L. rhamnosus* GR-1 treatment rescued the morphological abnormalities of dendritic spines in the hippocampus.

### *Lactobacillus rhamnosus* GR-1 supplementation restored specific microbiota changes in Pb-exposed rats

**FIGURE 3:**
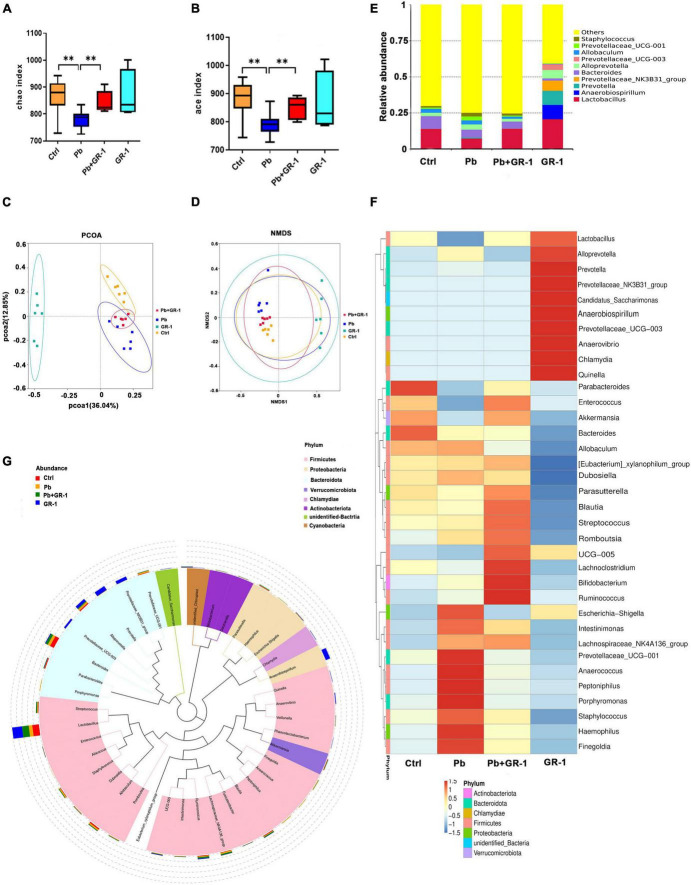
*Lactobacillus rhamnosus* GR-1 treatment restored specific microbiota changes in Pb-exposed rats. **(A,B)** Analysis of alpha diversity in gut microbiota by Chao1 and Ace analysis of the indicated groups (*n* = 6). **(C)** Principal co-ordinates analysis (PCOA) based on Bray–Curtis of gut microbiota of the indicated groups. **(D)** Non-metric multidimensional scaling (NMDS) analysis based on unweighted UniFrac metrics of gut microbiota of the indicated groups (NMDS = 0.068 < 0.2). **(E)** The stacked bar chart shows microbiota composition at the phylum level of the indicated groups. **(F,G)** Heatmap shows the relative abundance of representative microbiota at the genus level of the indicated groups. Abbreviations for microbial names are listed on the right of the graph. Ctrl, non-treated rats; Pb, lead-treated rats; Pb + GR-1, lead and GR-1-treated rats; GR-1, GR-1-treated rats. The data are represented as mean ± SEM; ^**^p < 0.01.

To figure out the microbial compositions involved in the *L. rhamnosus* GR-1 intervention, 16S rDNA sequencing was carried out using the IonS5TMXL sequencing platform. The Rarefaction Curve and Rank Abundance suggested that the total number of sequences and depth meets the requirement for sequencing and analysis (Supplementary Figures 4A,B). Microbial richness and evenness decreased after Pb exposure by Chao1 and Ace index. Compared to the Pb group, microbial richness was reversed by averages, from Chao1 and Ace analysis, as a consequence of *L. rhamnosus* GR-1 administration (Figures 3A,B). Principal co-ordinates analysis (PCOA) and non-metric multidimensional scaling (NMDS) analysis indicated the β-diversity among different microbial communities (Figures 3C,D). Based on this, Pb-treated rats displayed a dramatically distinct microbial composition from the control group. Upon probiotic *L. rhamnosus* GR-1 supplement, however, clustering of samples was distantly located from the Pb-treated samples and migrated to a similar profile with the healthy rats. Overall, it indicates that probiotic *L. rhamnosus* GR-1 administration restores, to an extent, the gut microbial diversity disrupted by chronic Pb exposure.

Pb exposure altered intestinal microbial abundance at the phylum level (Supplementary Figure 4C), which decreased the abundance of Bacteroidetes but increased the abundance of Firmicutes (Supplementary Figures 4D,E). However, there was no significant change in Bacteroidetes and Firmicutes phylum level after *L. rhamnosus* GR-1 treatment in Pb-exposed rats.

Subsequently, the relative abundance of specific bacteria was manifested at the genus level (Figure 3E). Pb exposure reduced the abundance of *Lactobacillus*, and *L. rhamnosus* GR-1 treatment led to higher proportions of *Lactobacillus* (Figures 3F,G). Besides, Pb exposure increased the abundance of *Staphylococcus*, *Prevotellaceae_–_UCG-001*, and *Alloprevotella*, while *L. rhamnosus* GR-1 treatment reversed the abundance of *Staphylococcus*, *Prevotellaceae-UCG-001*, and *Alloprevotella* abundance, indicating that *L. rhamnosus* GR-1 exerts a beneficial effect through optimization of microbiota architecture.

### *Lactobacillus rhamnosus* GR-1 repaired the Pb-induced learning and memory deficits *via* reshaping gut microbiota

**FIGURE 4:**
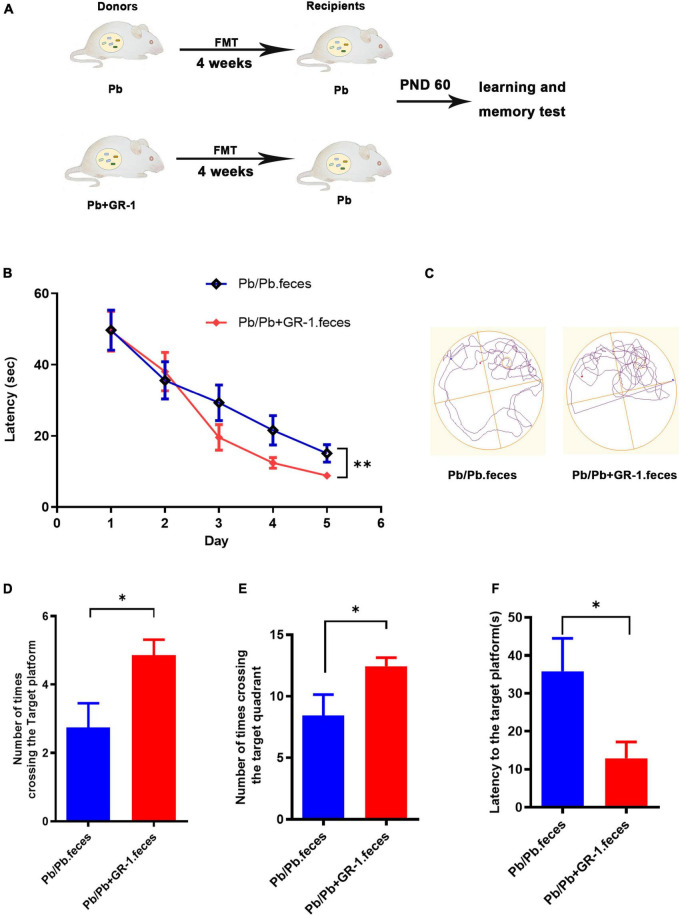
Reshaping gut microbiota with Pb + GR-1 feces repaired the Pb-induced learning and memory deficits. **(A)** Scheme of fecal microbiota transplantation (FMT) assay. **(B)** The escape latencies of MWM test over five consecutive training days. *n* = 8. **(C)** Representative swimming paths of indicated groups during the test day. **(D–F)** The average crossing number over the platform site **(D)**, the number of times crossing the target quadrant **(E)**, and the latency of the first target-site crossover during the test day **(F)**. *n* = 8. Data are shown as mean ± SEM. *p < 0.05, ^**^p < 0.01.

To identify whether *L. rhamnosus* GR-1 improved the Pb-induced memory deficits *via* reshaping the gut microbiome, colonization of Pb-exposed rats with the microbiota from Pb or Pb + GR-1 was performed, respectively (Figure 4A). According to the results of the MWM tests obtained during the training sessions, the latency to finding the target platform was shortened *via* the transplantation of feces from intervention donors of Pb + GR-1 rats (Figure 4B). On the test day, the rats administered with feces from the intervention donors of Pb + GR-1 rats crossed the zone of the removed platform more times than the rats administered with feces from the intervention donors of Pb rats (Figures 4C–F). Finally, these results demonstrated that *L. rhamnosus* GR-1 improved the injured memory of Pb-exposed SD rats *via* reshaping the gut microbiome.

### *Lactobacillus rhamnosus* GR-1 treatment alleviated intestinal damage of Pb-exposure rats

**FIGURE 5:**
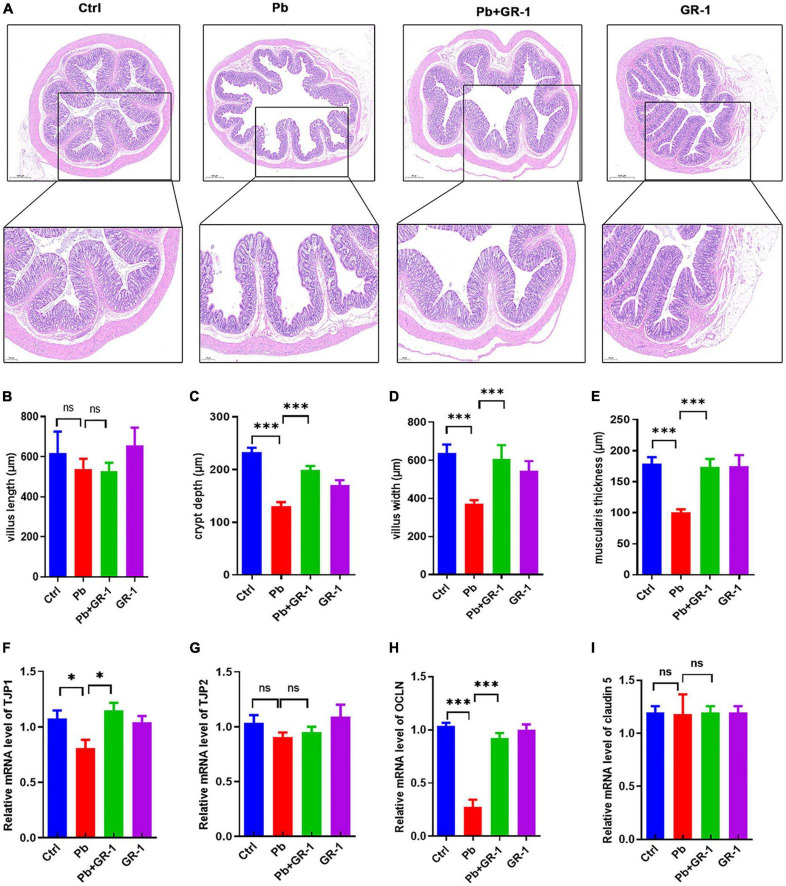
*Lactobacillus rhamnosus* GR-1 treatment alleviated intestinal damage of Pb-exposure rats. **(A)** The representative H&E staining images of colon tissue sections. *n* = 3. **(B–E)** The villus, crypt depth, villus width, and muscularis thickness of the colon in Ctrl, Pb, Pb + GR-1, and GR-1 groups. *n* = 3. **(F–I)** The mRNA levels of tight junction proteins in Ctrl, Pb, Pb + GR-1, and GR-1 groups, including TJP1, TJP2, OCLN, and claudin 5. *n* = 3 Data are shown as mean ± SEM. * *p* < 0.05, ^***^p < 0.001.

It is accepted that the integrity of the host’s intestinal barrier is damaged and rendered more permeable, which occurs when the gut epithelium tight junctions are impaired (21). Thus, we first tested whether *L. rhamnosus* GR-1 treatment could alleviate intestinal damage induced by Pb exposure. H&E staining of the colon reveals the interventional effect of *L. rhamnosus* GR-1 treatments on Pb-exposed rats (Figure 5A). The results found that the crypt depth, villus width, and muscularis thickness of Pb-exposed rats’ colon were reversed after supplementing *L. rhamnosus* GR-1, while there was no difference in the villus length of all groups (Figures 5B–E). Then, we investigated the mRNA levels of gut epithelium tight junction proteins, including TJP1, TJP2, OCLN, and claudin 5. The results found that Pb significantly reduced the mRNA level of TJP1 and OCLN, while *L. rhamnosus* GR-1 treatment alleviated the TJP1 and OCLN level in Pb-exposed groups (Figures 5F–I). Finally, we found that *L. rhamnosus* GR-1 alleviated the deficit in the intestinal barrier integrity of Pb-exposed rats.

### *Lactobacillus rhamnosus* GR-1 treatment reduced microgliosis and inflammation in the hippocampus of Pb-exposed rats

**FIGURE 6:**
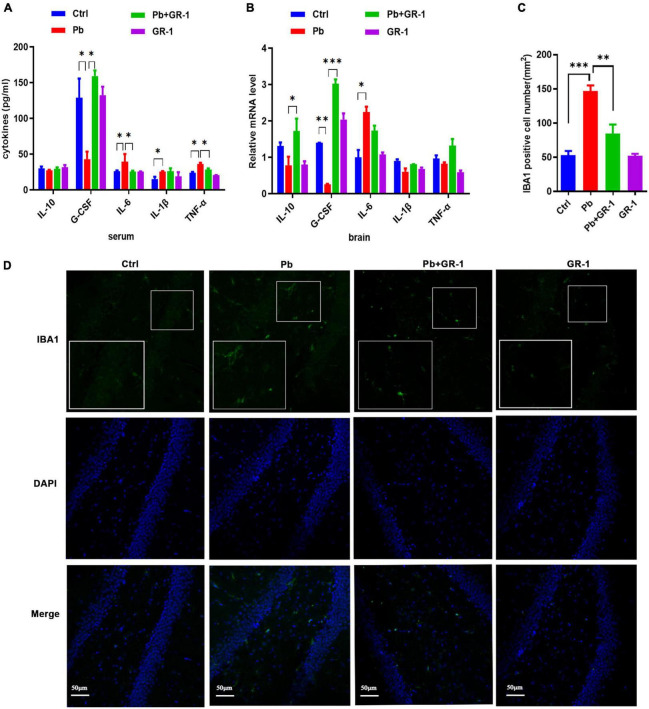
*Lactobacillus rhamnosus* GR-1 treatment reduced microgliosis and inflammation in the hippocampus of Pb-exposed rats. *n* = 6. **(A)** The serum cytokine expression of Ctrl, Pb, Pb + GR-1, and GR-1 groups, including IL-1β, IL-6, IL-10, TNF-α, and G-CSF. *n* = 6. **(B)** The mRNA expression levels of cytokines in the hippocampus, such as IL-1β, IL-6, IL-10, TNF-α, and G-CSF of the indicated groups. *n* = 6. **(C)** The number of IBA1-positive cells of each group. *n* = 3. **(D)** The immunohistochemistry staining was performed to detect IBA1 expression of the hippocampus in the indicated groups. *n* = 3. Data are shown as mean ± SEM. * *p* < 0.05, ^**^ *p* < 0.01, ^***^ *p* < 0.001.

Gut permeability is commonly associated with an altered immune response (22). To dissect the mechanisms through which *L. rhamnosus* GR-1 restores cognitive behavior, we mainly focused on inflammation and microgliosis. Accordingly, we assessed serum cytokines, including IL-1β, and IL-6, TNF-α, IL-10, and G-CSF. Results revealed that Pb exposure significantly increased the levels of TNF-α and IL-6, while *L. rhamnosus* GR-1 treatment decreased the levels of IL-6 and TNF-α. Besides, the enzyme-linked immunosorbent assay (ELISA) results found that Pb exposure decreased the G-CSF expression of anti-inflammatory cytokines, while *L. rhamnosus* GR-1 treatment unregulated the G-CSF expression of Pb-exposed rats (Figure 6A).

To test the consistency of inflammation between blood serum and brain, we assessed the hippocampus cytokines, including IL-1β, and IL-6, IL-10, TNF-α, and G-CSF (Figure 6B). Results revealed that Pb exposure significantly increased the levels of IL-6 in the hippocampus and GR-1 treatment decreased the levels of IL-6, while there was no aberrant difference between the Pb and Pb + GR-1 groups. In addition, the results found that Pb exposure decreased the IL-10 and G-CSF expression of anti-inflammatory cytokines, while *L. rhamnosus* GR-1 treatment unregulated the IL-10 and G-CSF expression of Pb-exposed rats.

Immunohistochemical examination with the microglia marker IBA1 of the brain revealed a significant increase in the number of microglia in Pb-exposed rats compared with the control groups, while *L. rhamnosus* GR-1 treatment decreased the number of microglia in the Pb-exposed rats (Figures 6C,D). These results indicated that *L. rhamnosus* GR-1 treatment attenuated Pb-induced learning and memory deficits *via* inhibiting microgliosis and inflammation.

### Granulocyte colony-stimulating factor treatment attenuated Pb-induced neurotoxicity *in vitro*

**FIGURE 7:**
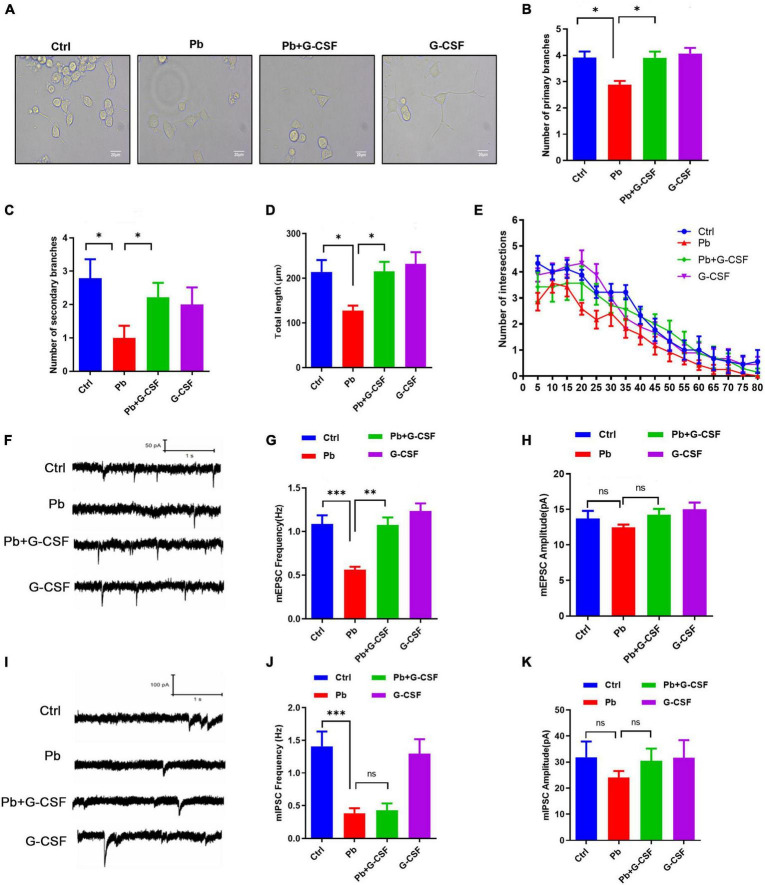
G-CSF treatment attenuates Pb-induced neurotoxicity. **(A–E)** Representative images of neurite outgrowth in PC12 cells upon various treatments **(A)** scale bar = 20 μm. The number of primary branches **(B)**, secondary branches **(C)**, total length **(D)**, and Sholl analysis **(E)** of neurite outgrowth of PC12 cells upon various treatments (∼50 cells). **(F–H)** Representative traces of miniature excitatory postsynaptic current (mEPSC) recording from cultured neurons treated with Pb, Pb + G-CSF, and G-CSF. The quantification of mEPSC frequency **(G)** and amplitude **(H)** from cultured neurons. **(I–K)** Representative traces of miniature inhibitory postsynaptic current (mIPSC) recording from cultured neurons treated with Pb, Pb + G-CSF, and G-CSF. The quantification of mIPSC frequency **(J)** and amplitude **(K)** from cultured neurons. Data are shown as mean ± SEM. *p < 0.05, ^**^p < 0.01, ^***^p < 0.001.

More recently, it has been shown that administration of G-CSF in stroke mouse models could induce neurogenesis near the damaged area of these rats, leading to neurological and functional recovery (23, 24). Besides, G-CSF could rescue the memory impairment of animal models of Alzheimer’s disease (25). Therefore, we next wondered whether G-CSF treatment, among the tested immune mediators (Figures 6A,B), could restore Pb-induced neurotoxicity *in vitro*. First, due to undifferentiated PC12 cell being a classic neural differentiation model, we investigated the role of G-CSF on the Pb-induced neurite outgrowth deficits in PC12 cells. Interestingly, the results found that 50 ng/ml G-CSF treatment for 48 h significantly ameliorated the Pb-induced neurite outgrowth deficits by reversing the number of primary branches, number of second branches, total length, and the number of intersections (Figures 7A–E).

Our previous studies showed that Pb exposure could inhibit synaptic transmission in cultured hippocampal neurons (18, 26). We next wondered whether G-CSF treatment restores excitatory and inhibitory synaptic transmission in cultured hippocampal neurons. The results found that 50 ng/ml G-CSF treatment for 48 h upregulated the Pb-induced the decrease of miniature excitatory postsynaptic current (mEPSC) frequency, not amplitude, indicating that 50 ng/ml G-CSF treatment restored the release of excitatory neurotransmitters (Figures 7F–H). However, there is no remarkable change in miniature inhibitory postsynaptic current (mIPSC) frequency and amplitude with or without 50 ng/ml G-CSF treatment of 5 μm Pb-exposed hippocampal neurons (Figures 7I–K). Taken together, these data revealed that G-CSF plays a critical role in Pb-induced neurotoxicity.

## Discussion

**FIGURE 8:**
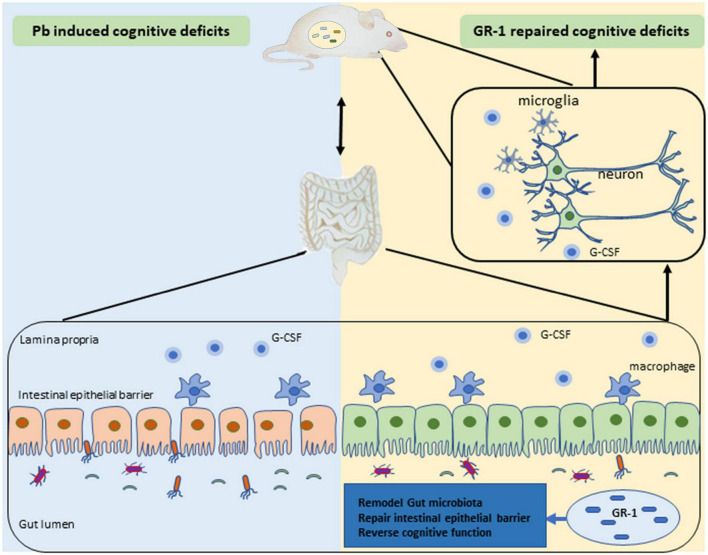
Schematic diagram of *Lactobacillus rhamnosus* GR-1 attenuated the Pb-induced learning and memory deficits.

In this article, we studied the effects of probiotics supplementation on the learning and memory ability of Pb-exposed rats. Administration of *L. rhamnosus* GR-1 but not *L. reuteri* RC-14 or *L. rhamnosus* LGG reversed Pb-induced cognitive deficits in rats exposed to Pb, suggesting that the modulation of cognitive function varies among *Lactobacillus* species. We also found that *L. rhamnosus* GR-1 modulated the gut microbiota and formed characteristic microbial communities, leading to better maintenance of the mucosal barrier. Besides, *L. rhamnosus* GR-1 supplementation inhibited hippocampal microgliosis and proinflammatory cytokine expression of Pb-exposed rats (Figure 8).

Compelling evidence suggests an association between Pb exposure and gut intestinal barrier injury (5). It was reported that 6-week-old male C57BL/6 mice were treated with 0.2-ml Pb solution at 20, 100, 500, or 1,000 mg Pb/kg BW/day for 3 days, leading to increased tissue damage and inflammation in the mouse colon in a dose-dependent manner (5). Consistent with our findings, chronic Pb exposure for 2 weeks also impaired the colon of the chronic Pb-exposed rats. Besides, Pb exposure induced a decrease in the TJP1 and OCLN levels, which has harmful effects on the gut barrier. Therefore, the damage of Pb exposure to intestinal structural integrity is established.

The effects of Pb on the composition of the gut microbiota have been reported in many previous studies (27, 28), which is also a barrier that prevents ingested harmful metabolite from affecting intestinal cells and limits their toxicity. Here, we found that the relative abundances of Firmicutes and Bacteroidetes were significantly altered, indicating the Pb-induced disorder in the gut microbiota. Besides, in our study, the relative abundances of *Lactobacillus* were significantly decreased after Pb exposure; however, in one study, it was found that the relative abundance of *Lactobacillus* increased after Pb exposure at 1,000 mg Pb/kg BW/day for 3 days. This different phenomenon may be explained by alterations in the time and dose of Pb exposure.

At present, many studies have found that probiotics have the function of excreting heavy metals (29, 30). However, studies on the therapeutic effects of probiotics on chronic heavy metal Pb exposure, which is a more important and common pattern of brain damage, are limited. Therefore, this study mainly focuses on improving memory after chronic Pb injury with long-term probiotic intervention through the remolding gut–brain axis. It was reported that *L. rhamnosus* GR-1 can mediate heavy metal sequestration on Pb and Cd absorption *in vitro* (30), but we confirmed that the *L. rhamnosus* GR-1 used in this study failed to reduce the concentration of Pb in the blood. These discrepancies might result from Pb exposure, acute and chronic exposure, and the concentration of Pb. In addition, it is less likely that the direct combination of Pb and *L. rhamnosus* GR-1 *in vivo* is due to the integrity of the intestinal barrier.

Several control experiments underscore the specificity of *L. rhamnosus* GR-1-mediated rescue of hippocampal-dependent cognitive function in Pb-exposed rats. First, treatment with heat-killed *L. rhamnosus* GR-1 or *L. rhamnosus* GR-1 supernatant failed to restore hippocampal-dependent cognitive function in Pb-exposed rats. Second, similar treatment with another bacteria, *L. rhamnosus* GG, or *L. reuteri* RC-14 failed to rescue hippocampal-dependent cognitive function, which, in part, is because the protective effects are strain-specific. Hence, identification of the full spectrum of active ingredients in *L. rhamnosus* GR-1 warrants further investigative attention.

It has been reported that activated microglial cells release proinflammatory cytokines TNF-α, IL-1β, and IL-6, which negatively regulate cognitive functions (31, 32). Hippocampal inflammation is highly associated with recognition memory impairment. Our findings demonstrated that *L. rhamnosus* GR-1 attenuated Pb-induced activation of microglia and altered the expression of inflammatory cytokines. Therefore, *L. rhamnosus* GR-1 treatment that inhibits hippocampal microgliosis and inflammation may contribute to spatial learning and memory restoration.

*Lactobacillus rhamnosus* is a human commensal with known immunomodulatory properties. It was reported that the production of G-CSF by a protein-like *L. rhamnosus* GR-1 secretory factor by activating TLR2-dependent signaling in macrophages is preferred (33, 34). Additionally, a study found that *L. rhamnosus* GR-1 potently induced production of G-CSF, which was a crucial mediator for suppressing TNF-α production in macrophages *in vitro* (34). In our study, *L. rhamnosus* GR-1 supplementation upregulated the G-CSF expression and treatment of G-CSF attenuated the Pb-induced neurotoxicity *in vitro*. It is worth noting that G-CSF is not the only key molecular regulating the cognitive deficits due to Pb exposure, which was partly demonstrated by the consistent performance of IL-6 and TNF-α cytokines, as well as the ensuing crucial incidents of microgliosis.

Finally, our data demonstrated that probiotics *L. rhamnosus* GR-1 have a protective role against the deleterious effects of Pb exposure on gut microbiota, immune cell cytokines, and eventually cognitive deficits. Further characterization of the *L. rhamnosus* GR-1 will clarify the protective effect of probiotics on cognitive defects and promote the development of a new therapeutic strategy for Pb-induced neurotoxicology.

## Data Availability Statement

The datasets generated during microbiome analysis are available in the Mendeley repository, Mendeley Data, V1, doi: 10.17632/ykv4p3wkfh.1. The direct link of the sequencing data: (https://data.mendeley.com/datasets/ykv4p3wkfh/1). Further information and requests for resources and reagents should be directed to and will be fulfilled by the corresponding author, H-LW (wanghl@hfut.edu.cn).

## Ethics statement

The animal study was reviewed and approved by the Institutional Animal Care and Use Committee of Hefei University of Technology, China.

## Author contributions

XG and H-LW designed the experiments. XG performed the Western blot, Q-PCR, Golgi–Cox staining, and cell culture. NB and CH performed primary neuronal culture electrophysiological recording. XG, TW, and RW performed the MWM behavior experiments. XG and YX wrote the manuscript. H-LW supervised the work. All authors approved the final version of the manuscript.

## Conflict of Interest

The authors declare that the research was conducted in the absence of any commercial or financial relationships that could be construed as a potential conflict of interest.

## Publisher’s Note

All claims expressed in this article are solely those of the authors and do not necessarily represent those of their affiliated organizations, or those of the publisher, the editors and the reviewers. Any product that may be evaluated in this article, or claim that may be made by its manufacturer, is not guaranteed or endorsed by the publisher.
