# Supplementary material for: Probiotic Lactobacillus rhamnosus GR-1 supplementation attenuates Pb-induced learning and memory deficits by reshaping the gut microbiota
Source: Front Nutr. 2022 Jul 19;9:934118. doi: 10.3389/fnut.2022.934118 (PMC9344877; doi:10.3389/fnut.2022.934118)
Supplement: Supplementary file 1 [file Data_Sheet_1.docx]

**Supplementary Information**

**Probiotic Lactobacillus rhamnosus GR-1 supplementation attenuates Pb-induced learning and memory deficits by reshaping the gut microbiota**

Xiaozhen Gu^1, 2^, Nanxi Bi^1, 2^, Tian Wang^1, 2^, Chengqing Huang^1, 2^, Rongrong Wang^1, 2^, Yi Xu^1, 2, *^, Hui-Li Wang^1, 2, *^

1. Engineering Research Center of Bio-process, Ministry of Education, Hefei University of Technology, 193 Tunxi Road, Hefei, Anhui 230009, PR China.

2. School of Food and Biological Engineering, Hefei University of Technology, No. 193 of Tunxi Road, Baohe District, 230009, Hefei, China.

*Corresponding author: Yi Xu, School of Food and Biological Engineering, Hefei University of Technology, No. 193 of Tunxi Road, Baohe District, 230009, Hefei, China. E-mail: xuyixuyi3734@163.com

Hui-Li Wang, School of Food and Biological Engineering, Hefei University of Technology, No. 193 of Tunxi Road, Baohe District, 230009, Hefei, China. Tel & Fax: +86 62919397; E-mail: [wanghl@hfut.edu.cn](mailto:wanghl@hfut.edu.cn), Tel: +86 551 62919397, Fax: +86 551 62919397

Figure S1. The average weight of the indicated groups during the developmental stages.


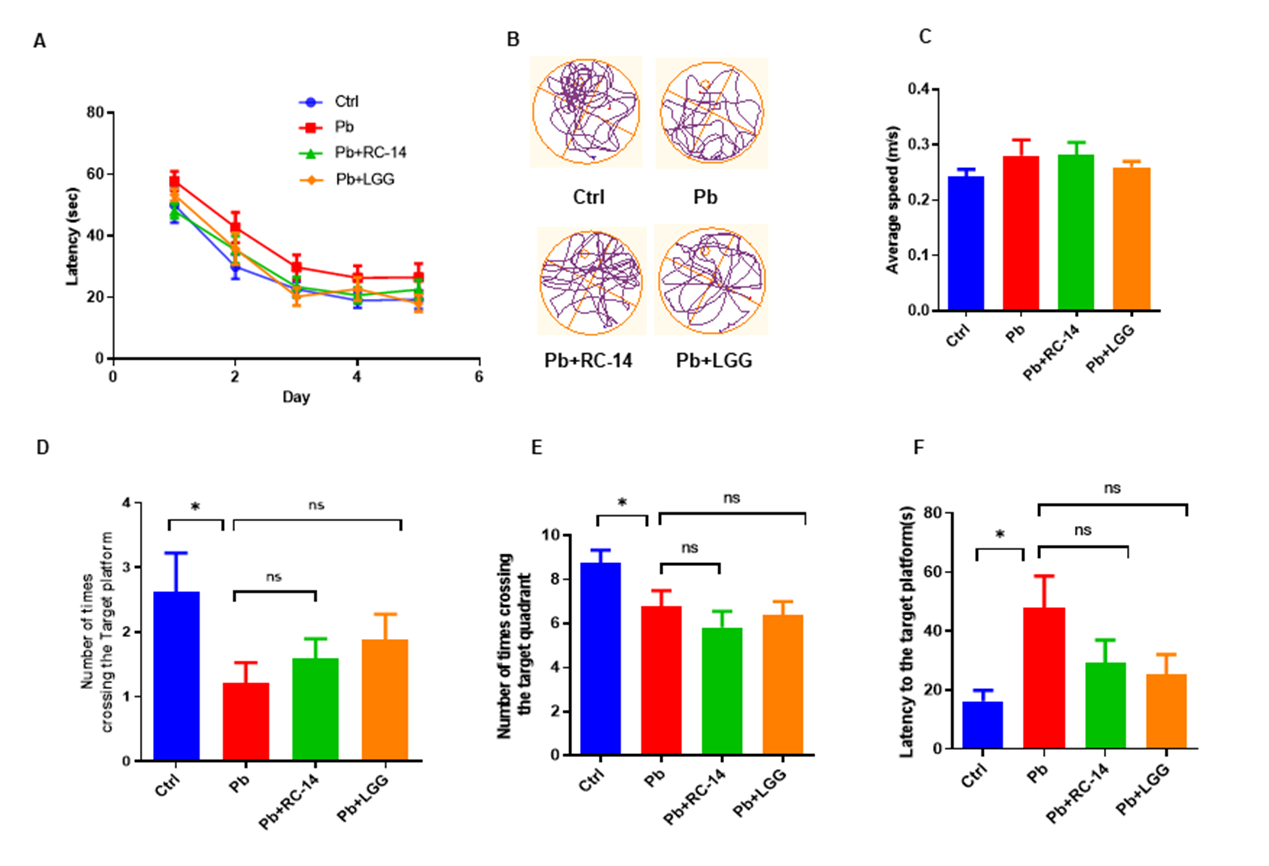


Figure S2. The effects of Administration of LGG and RC-14 on Pb-induced learning and memory deficits. (A) The escape latencies in the four groups over five consecutive training days. (B) Representative swimming paths of Ctrl, Pb or Pb+GR-1, and GR-1 during the test day. (C) The average speed of each group during the MWM test. (D-F) The average crossing number over the platform-site (D), the number of times crossing the target quadrant (E), and the latency of the first target-site crossover (probe time) during the probe trial (F). Data were shown as mean ± SEM. *: *p* < 0.05, **: *p* < 0.01, ***: *p* < 0.001.


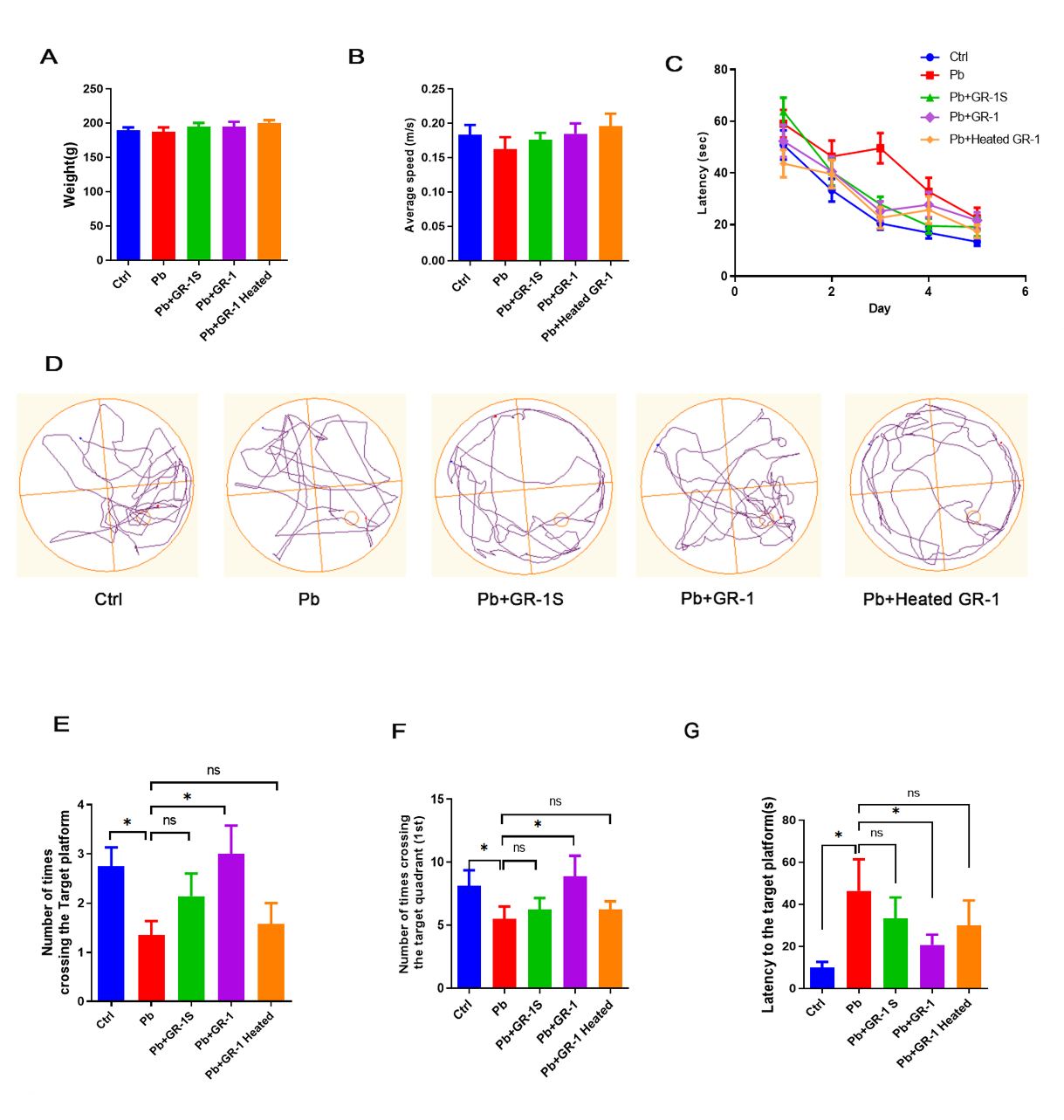


Figure S3. The effects of Administration of GR-1 supernatant and heated killed-GR-1 on Pb-induced learning and memory deficits. (A) The average weight of the indicated groups in 2 months after birth. (B) The average speed of each group during the MWM test. (C) The escape latencies in the four groups over five consecutive training days. (D) Representative swimming paths of Ctrl, Pb or Pb+GR-1, and GR-1 during the test day. (E-G) The average crossing number over the platform-site (E), the number of times crossing the target quadrant (F), and the latency of the first target-site crossover (probe time) during the probe trial (G). Data were shown as mean ± SEM. *: *p* < 0.05, **: *p* < 0.01, ***: *p* < 0.001.


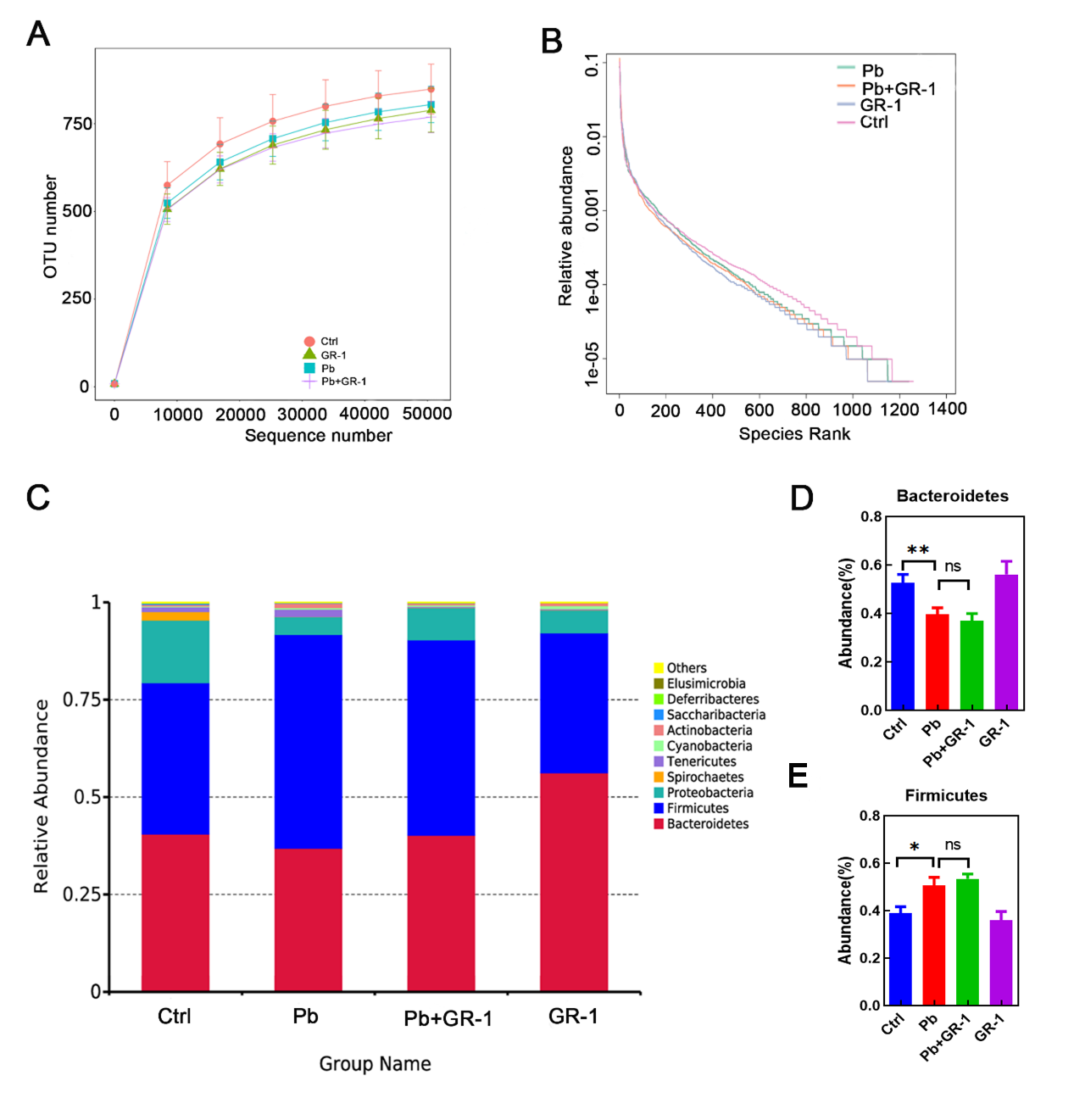


Figure S4. Effects of GR-1 on the gut microbiome structure in Pb-exposed rats, related to Figure 3. (A) Rarefaction Curve of each group. n=6 (B) Rank Abundance each group. (C-D) Shannon index (C) and Simpson index. n=6 (D). (E) Relative Bacteroidetes abundance of the gut microbiota in phylum levels. n=6 (F) Relative Fimicutes abundance of the gut microbiota in phylum levels. n=6 (E) Relative *Fimicutes*/*Bacteroidetes* abundance of the gut microbiota in phylum levels. n=6

Table S1 Primers used in the study

| gene | Forward primer（5’-3’） | Reverse primer（5’-3’） |
| --- | --- | --- |
| G-CSF | ACCTACAAGCTGTGTCATCCG | CTCAGGCACTTTGTCTGCTG |
| IL-10 | TATGTTGCCTGCTCTTACTGGC | TGTCAGCAGTATGTTGTCCAGC |
| IL-1β | AGCATCTCGACAAGAGCTTCAG | ATCATCCCACGAGTCACAGAG |
| IL-6 | TGCTCTGGTCTTCTGGAGTTCC | TTGGAAGTTGGGGTAGGAAGG |
| TNF-α | TACTGAACTTCGGGGTGATCGG | CTTGGTGGTTTGCTACGACG |
| TJP1 | ATGGTCTTCGATTGGCCAGC | TAGCCCGCTCATCTCTTTGCAC |
| TJP2 | ACATCACAGCTGCAGGTGTC | TCACCATTTTGGTGTTAGGGCC |
| OCLN | GGACTGGCTCAGGGAATATCCA | AGCCATGTACTCTTCGCTCTCC |
| claudin5 | TGCCTTCCTGGACCACAATATC | TAACAAAGAGTGCCACAAGCG |
